# Cardiac arrest induced by the intentional ingestion of boric acid and mirtazapine treated by percutaneous cardiopulmonary bypass: a case report

**DOI:** 10.1186/s13256-019-2079-7

**Published:** 2019-05-16

**Authors:** Hiroki Nagasawa, Hiroaki Nakanishi, Kazuyuki Saito, Takehisa Matsukawa, Kazuhito Yokoyama, Youichi Yanagawa

**Affiliations:** 1 Department of Acute Critical Care Medicine, Shizuoka Hospital, Juntendo University, 1129 Nagaoka, Izunokuni City, Shizuoka 410-2295 Japan; 2 Department of Forensic Medicine, Juntendo University, Tokyo, Japan; 3 Department of Epidemiology and Environmental Health, Juntendo University, Tokyo, Japan

**Keywords:** Boric acid, Mirtazapine, Percutaneous cardiopulmonary bypass

## Abstract

**Background:**

Mirtazapine has a good tolerability and safety profile that demonstrates several benefits over other antidepressants and it is associated with few fatalities. Boric acid is an odorless white powder that is generally not recognized as a poisonous substance. We report a case of cardiac arrest induced by the intentional ingestion of mirtazapine, boric acid, and sennosides, by a patient who required percutaneous cardiopulmonary bypass.

**Case presentation:**

Our patient was a 49-year-old Japanese woman with a history of depression; she was found in an unconscious state after ingesting boric acid (unknown amount), mirtazapine (1950 mg), and sennosides (780 mg). On arrival, she was in a deep coma with marked hypotension induced by atrial fibrillation, tachycardia, and diffuse hypokinetic cardiac motion. She had systemic diffuse erythema. Her serum concentrations of boric acid and mirtazapine on arrival were 560.49 mg/L and 1270 ng/mL, respectively. She experienced repeated cardiac arrest, and was therefore treated with tracheal intubation, mechanical ventilation, percutaneous cardiopulmonary bypass, and continuous hemodialysis filtration. Stable circulation and respiration and a normal kidney function were finally obtained and she was transferred to a local medical facility in a persistent unconscious state.

**Conclusions:**

This is the first case of a return of spontaneous circulation after cardiac arrest induced by the intentional ingestion of boric acid and mirtazapine, requiring percutaneous cardiopulmonary bypass for survival. To maintain cerebral perfusion during percutaneous cardiopulmonary bypass, even in a prolonged state of cardiac arrest induced by overdose, is medically, ethically, and economically challenging.

## Background

Mirtazapine is a noradrenergic and specific serotoninergic antidepressant agent that stimulates the release of norepinephrine and serotonin while also blocking serotonin receptors, that is, 5-hydroxytryptamine_2_ (5-HT_2_) and 5-hydroxytryptamine_3_ (5-HT_3_) [1]. Mirtazapine has a good tolerability and safety profile that demonstrates several benefits over other antidepressants and it is associated with few fatalities [1, 2]. Boric acid is an odorless white powder that is generally not recognized as a poisonous substance. However, if ingested in massive quantities, boric acid can have potentially fatal effects, including metabolic acidosis, acute renal failure, and heart failure [3, 4].

Percutaneous cardiopulmonary bypass or extracorporeal membrane oxygenation is an external approach that supports the cardiopulmonary system by providing oxygenation and ensuring the cardiac function of patients with cardiac and respiratory failure. Percutaneous cardiopulmonary bypass has been successfully used in patients of all ages with various medical and surgical conditions leading to cardiovascular collapse, respiratory failure, cardiogenic shock, or refractory hypotension [5]. Extracorporeal membrane oxygenation has also been used in cases of poisoning when cardiac arrest or refractory hypotension develops, although such instances are rare [5].

We feel it is important to accumulate more reports on toxicology cases treated with percutaneous cardiopulmonary bypass [5–12]. Accordingly, we report a case of cardiac arrest induced by the intentional ingestion of mirtazapine, boric acid, and sennosides by a patient who required percutaneous cardiopulmonary bypass.

## Case presentation

Local Institutional Review Board approval was obtained, and the patient’s guardian gave their written, informed consent to publish this case.

A 49-year-old Japanese woman with a history of depression was found in an unconscious state by her husband after ingesting an unknown amount of boric acid, mirtazapine (1950 mg), and sennosides (780 mg) and transported to a local medical facility. She had unstable circulation so she was transported to our Department of Acute Critical Care Medicine. On arrival, her vital signs were as follows: Glasgow Coma Scale, E1V1M1; blood pressure, 45/13 mmHg; heart rate, 190 beats per minute; and body temperature, 37.0 °C. She had systemic diffuse erythema. There were no other physical findings. Electrocardiography (ECG) showed atrial fibrillation tachycardia. She received tracheal intubation with mechanical ventilation. A cardiac echocardiogram depicted diffuse hypokinesis of wall motion with an ejection fraction of 20%. Chest roentgenography revealed no findings. Whole-body computed tomography to evaluate her brain condition, residual drugs in her stomach, and accompanying lesions indicated bilateral dorsal lung consolidation, suggesting aspiration [13]. The results of an arterial blood gas analysis, cell blood count, and biochemical study are shown in Table 1. Soon after, she exhibited pulseless electrical activity; spontaneous circulation was obtained by advanced life support. However, she experienced repeated episodes of pulseless electrical activity, and percutaneous cardiopulmonary bypass was required due to unstable circulation. An emergency coronary angiogram was negative. She was admitted to an intensive care unit and underwent additional continuous hemodialysis filtration due to acute kidney injury with anuria. She remained in a deep coma state without sedation. On the second hospital day, cardiac motion ceased on the echocardiogram, but her atrial fibrillation rhythm continued. As her husband strongly wished to continue these treatments, we selected to continue them until cardiac standstill. However, cardiac motion was obtained again on the fourth hospital day and stable spontaneous circulation with sinus rhythm was obtained on the sixth hospital day; thus, percutaneous cardiopulmonary bypass was withdrawn. She exhibited hair loss. She remained in a persistent unconscious state; thus, tracheostomy was performed on the seventh hospital day. Sufficient urinary flow was obtained, and her potassium level remained within the normal limits; thus, continuous hemodialysis filtration was withdrawn on the tenth hospital day. Head magnetic resonance imaging showed signal change in the bilateral white matter and caudate nuclei, suggesting hypoperfusional cerebral ischemia. As she developed right leg dry necrosis, which was induced by cannulation for percutaneous cardiopulmonary bypass, leg amputation was performed on the 22nd hospital day. She also developed bilateral corneal ulcers, which was possibly due to boric acid poisoning.

**Table 1:** The results of the laboratory analysis

Arterial blood gas (10 L/minute oxygen)			
pH	7.499	PCO_2_	41.6 mmHg,
PO_2_	429 mmHg	HCO_3_^−^	32.1 mmol/l
Base excess	8.4 mmol/l	Lactate	10.7 mmol/l
Cell blood count			
White blood cell count	12,900/μl	Hemoglobin	6.0 g/dl
Red blood cell count	330 × 10^4^/μl	Hematocrit	20%
Platelet count	37.8 × 10^4^/μl		
Serum biochemistry			
Total protein	6.3 g/dl	Albumin	3.3 g/dl
Total bilirubin	0.5 mg/dl	Cholinesterase	176 IU/L
Aspartate aminotransferase	20 IU/l	Alanine aminotransferase	7 U/l
γ-glutamyl transpeptidase	9 IU/L	Alkaline phosphatase	159 IU/L
Creatine phosphokinase	307 IU/l	Amylase	74 IU/L
Blood urea nitrogen	32 mg/dl	Creatinine	3.0 mg/dl
Glucose	168 mg/dl	HbA_1_C	5.8%
Sodium	152 mEq/l	Potassium	2.5 mEq/l
Chloride	104 mEq/l	C reactive protein	1.1 mg/dl
Ammonia	53 μg/dL		
Coagulation			
Activated partial thromboplastin time	29 (24.9) seconds		
Prothrombin time	21 (12.1) seconds		
Fibrinogen	195 mg/dl		
Fibrinogen degradation products	3.3 μg/mL		

*HbA*_*1*_*C* glycated hemoglobin, *HCO*_*3*_^*-*^ bicarbonate, *pCO*_*2*_ partial pressure of carbon dioxide, *pO*_*2*_ partial pressure of oxygen

**Fig. 1:**
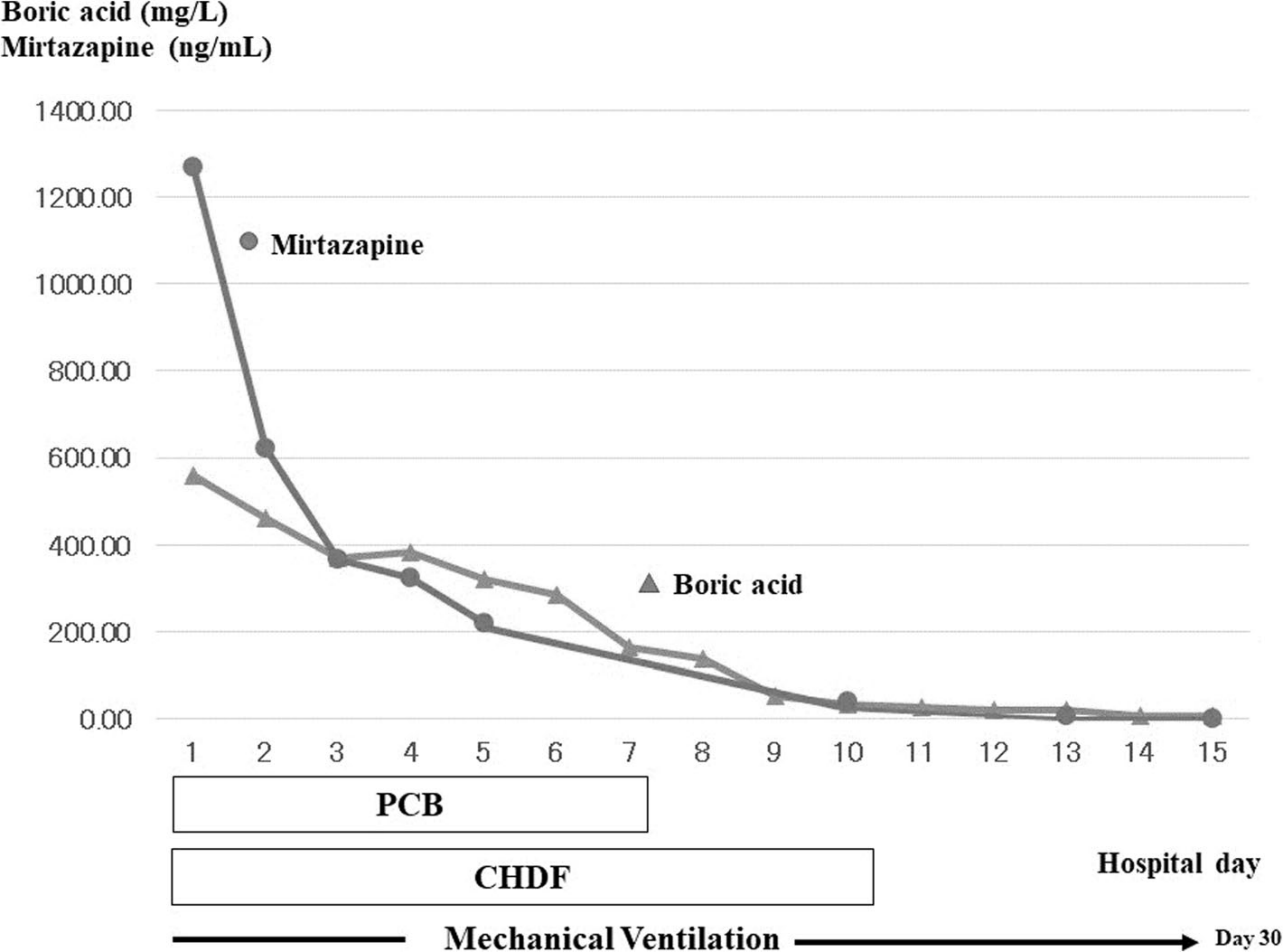
The time course of the serum concentrations of boric acid and mirtazapine. The initial levels of boric acid and mirtazapine were 560.49 mg/L and 1270 ng/mL, respectively. *CHDF* continuous hemodiafiltration, *PCB* percutaneous cardiopulmonary support

Stable spontaneous respiration was finally obtained on the 30th hospital day; thus, mechanical ventilation was withdrawn. Systemic erythema gradually subsided with desquamation. She displayed spontaneous eye opening and spontaneous movement of her extremities; however, she could not respond to any commands. She was transferred to a local medical facility in a persistent unconscious state on the 39th hospital day. A subsequent examination revealed that her serum concentrations of boric acid and mirtazapine on arrival were 560.49 mg/L and 1270 ng/mL, respectively (Fig. 1). She could speak simple words for commands and swallow food but was unable to walk; thus, at 6 months after collapse, she was totally dependent in her activities of daily living.

## Discussion

This is the first case of cardiac arrest induced by the intentional ingestion of boric acid and mirtazapine, in which percutaneous cardiopulmonary bypass was required for the patient to survive.

With regard to cardiotoxicity, Leonard *et al.* [14] investigated the association between exposure to antidepressants and emergency department or in-patient admission for sudden cardiac death and ventricular arrhythmia (SD/VA). Among 1.3 million person-years of antidepressant exposure, they identified 4222 SD/VA outcomes, which amounted to a rate of 3.3/1000 person-years (95% CI, 3.2–3.4). In comparison to paroxetine (a referent with a reportedly favorable cardiovascular risk profile), the adjusted hazard ratio (HR) for mirtazapine was 1.26 (1.11–1.42). They concluded that of the antidepressants that were studied, only mirtazapine had a statistically significantly greater SD/VA risk than paroxetine. A massive ingestion of boric acid generally induces metabolic acidosis, renal failure, and skin disturbance. However, boric acid can also induce lethal arrhythmia and cardiac failure [3, 4]. Accordingly, both boric acid and mirtazapine could have caused fatal cardiac arrest due to direct cardiac intoxication in this case. Regarding the toxic effects of sennosides in laboratory animals, the lethal dose 50 value was approximately 5000 mg/kg, and the cause of death was extensive water and electrolyte loss following massive diarrhea [15]. However, the patient in the present case did not ingest a fatal dose and dehydration was not observed; thus, the possibility that sennosides were involved in the patient’s cardiac arrest could be denied.

Percutaneous cardiopulmonary bypass may be helpful in cases of exposure to multiple serious toxicological agents that results in temporary cardiorespiratory failure or metabolic dysfunction. Percutaneous cardiopulmonary bypass alone does not remove or neutralize any toxins but does provide hemodynamic support and oxygenation until the toxins can be eliminated or end-organ recovery is achieved. A case series of 62 patients in France showed similar survival rates of 76% in patients receiving percutaneous cardiopulmonary bypass due to severe acute drug intoxication, with a lower overall mortality rate than in patients who received supportive care alone [16, 17]. The cardiotoxic effects of antidepressants are reported to be temporary, and there have been some cases in which patients who have exhibited unstable circulation due to overdose have also achieved social rehabilitation after percutaneous cardiopulmonary bypass treatment [18, 19]. The present case also demonstrated that the cardiotoxicity induced by the massive ingestion of mirtazapine and boric acid was a temporary effect.

However, whether or not percutaneous cardiopulmonary bypass can improve the survival in patients with cardiac arrest due to toxicological agent exposure remains unclear. Attempting to initiate percutaneous cardiopulmonary bypass during cardiac arrest is difficult, as it may require pausing cardiopulmonary resuscitation in order to cannulate and initiate the procedure, which may result in an unfavorable outcome. In addition, thoracic cage injury induced by chest compression may result in fatal complications due to coagulopathy after the initiation of percutaneous cardiopulmonary bypass, requiring heparinization [20]. Furthermore, our attempt to use percutaneous cardiopulmonary bypass to obtain social rehabilitation failed due to hypoperfusional cerebral ischemia induced by prolonged cardiac arrest, which has also been reported in patients who underwent percutaneous cardiopulmonary bypass after experiencing cardiac arrest triggered by other causes [21].

The indication of percutaneous cardiopulmonary bypass for poisoned patients remains a major problem. First, substantial resources and costs as well as a multidisciplinary team including toxicologists, intensivists, and surgeons, are required to perform and manage percutaneous cardiopulmonary bypass, so few facilities are equipped to activate it in a timely fashion [5]. Second, percutaneous cardiopulmonary bypass is associated with a number of potential complications, including limb ischemia, compartment syndrome, cerebral ischemia, acute kidney injury, bleeding, emboli, and infection [5]. Third, several factors must be considered on an individual basis, such as the patient’s age, comorbidities, risk for complications, survivability, specific drug or chemical involved in the exposure, and time of hypoperfusion or cardiac arrest [5]. Accordingly, further studies are needed in order to establish criteria for considering percutaneous cardiopulmonary bypass in poisoned patients.

## Conclusion

This is the first case in which a return of spontaneous circulation was obtained after cardiac arrest induced by the intentional ingestion of boric acid and mirtazapine, in a patient who required percutaneous cardiopulmonary bypass for survival. The present case demonstrated that the cardiotoxicity induced by the massive ingestion of mirtazapine and boric acid was a temporary effect. To maintain cerebral perfusion during percutaneous cardiopulmonary bypass, even in cases involving prolonged cardiac arrest induced by overdose, is medically, ethically, and economically challenging.

## Abbreviations

5-HT_2_: 5-Hydroxytryptamine_2_
5-HT_3_: 5-Hydroxytryptamine_3_
ECG: Electrocardiography
HR: Hazard ratio
SD/VA: Sudden cardiac death and ventricular arrhythmia
